# A Non-Canonical Calmodulin Target Motif Comprising a Polybasic Region and Lipidated Terminal Residue Regulates Localization

**DOI:** 10.3390/ijms21082751

**Published:** 2020-04-15

**Authors:** Benjamin M. M. Grant, Masahiro Enomoto, Mitsuhiko Ikura, Christopher B. Marshall

**Affiliations:** 1 Princess Margaret Cancer Centre, University Health Network, 101 College St., Toronto, ON M5G 1L7, Canada; Benjamin.Grant@uhnresearch.ca; 2 Department of Medical Biophysics, University of Toronto, 101 College St., Toronto, ON M5G 1L7, Canada

**Keywords:** Calmodulin, KRAS4b, prenylation, myristoylation, polybasic region, Ca^2+^ signaling, protein structure/function, membrane association

## Abstract

Calmodulin (CaM) is a Ca^2+^-sensor that regulates a wide variety of target proteins, many of which interact through short basic helical motifs bearing two hydrophobic ‘anchor’ residues. CaM comprises two globular lobes, each containing a pair of EF-hand Ca^2+^-binding motifs that form a Ca^2+^-induced hydrophobic pocket that binds an anchor residue. A central flexible linker allows CaM to accommodate diverse targets. Several reported CaM interactors lack these anchors but contain Lys/Arg-rich polybasic sequences adjacent to a lipidated N- or C-terminus. Ca^2+^-CaM binds the myristoylated N-terminus of CAP23/NAP22 with intimate interactions between the lipid and a surface comprised of the hydrophobic pockets of both lobes, while the basic residues make electrostatic interactions with the negatively charged surface of CaM. Ca^2+^-CaM binds farnesylcysteine, derived from the farnesylated polybasic C-terminus of KRAS4b, with the lipid inserted into the C-terminal lobe hydrophobic pocket. CaM sequestration of the KRAS4b farnesyl moiety disrupts KRAS4b membrane association and downstream signaling. Phosphorylation of basic regions of N-/C-terminal lipidated CaM targets can reduce affinity for both CaM and the membrane. Since both N-terminal myristoylated and C-terminal prenylated proteins use a Singly Lipidated Polybasic Terminus (SLIPT) for CaM binding, we propose these polybasic lipopeptide elements comprise a non-canonical CaM-binding motif.

## 1. Introduction

Calmodulin (CaM) is an exceptionally highly conserved 16.7 kDa acidic protein that senses Ca^2+^ and interacts with and regulates a wide variety of target proteins including enzymes, kinases and phosphatases, ion channels and pumps, and transcription factors [1].

**Figure 1:**
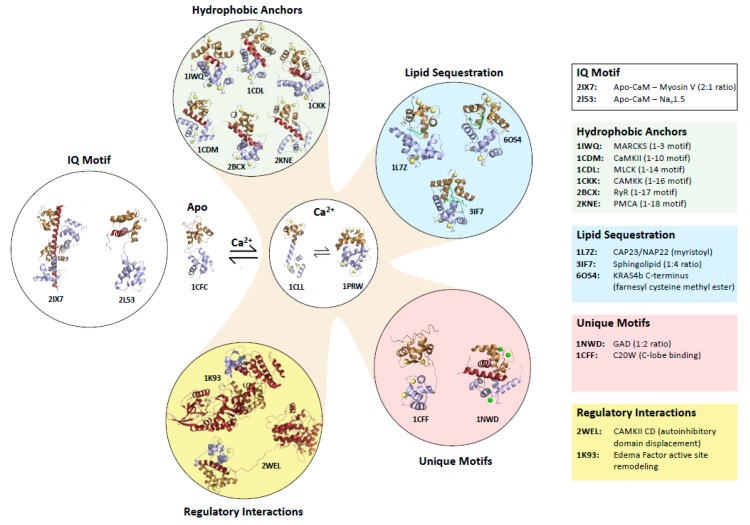
Diversity of calmodulin target binding. Structures of canonical and non-canonical calmodulin:target complexes selected to illustrate diverse modes of binding. PDB accession codes for each structure are indicated along with the target name and motif type. The calmodulin C-terminal and N-terminal lobes are coloured brown and blue, respectively, and target peptides and proteins are dark red. Lipid components of targets are green. Acronyms are: MARCKS (myristoylated alanine-rich C kinase substrate), CaMKII (Calmdoulin Kinase II), MLCK (Myosin Light Chain Kinase), CaMKK (Calmodulin Kinase Kinase), RyR (Ryanodine Receptor), PMCA (Protein Misfolding Cyclic Amplification), CaMKII CD (CaMKII C-terminal domain), C20W (the C20W peptide from the plasma membrane calcium pump), GAD (Glutamate Decarboxylase). CaM:target stoichiometry is indicated in cases where it is not 1:1 (e.g., one molecule of CaM binds two GAD or four sphingolipids, and two CaM peptides bind a single myosin peptide).

The 148-amino acid sequence of CaM contains four Ca^2+^-binding EF-hands, each of which is a 29-a.a. helix-loop-helix motif. N-terminal and C-terminal lobes of CaM comprise a pair of EF-hands and form a globular domain with exposed hydrophobic surfaces when bound to Ca^2+^ ions. The two lobes are connected by a flexible ‘hinge’ region (^78^DTDS^81^) that allows the protein to adopt a range of conformations with a variety of orientations of the lobes [2,3,4] (Figure 1). Ca^2+^ binding induces a conformational change in each lobe, which leads to the exposure of methionine-rich hydrophobic pockets that engage hydrophobic side chains of target proteins. The flexible side chain of methionine together with the flexible linker confers the ability of CaM to recognize a wide variety of target proteins with distinct structural features [2,5,6,7].

### 1.1. Target Protein Binding Motifs of Ca^2+^-Bound and apo-CaM

While many CaM interactions with target proteins are dependent on Ca^2+^ binding, some occur in the absence of Ca^2+^ and there are targets that are recognized by both Ca^2+^-bound and Ca^2+^-free states [1]. The recognition sites in CaM targets are diverse in sequence, but often form amphipathic helices made from basic and hydrophobic residues amino acids. Ca^2+^-dependent recognition of a CaM target protein is generally mediated by hydrophobic interactions between each of the two lobes and two hydrophobic side chains referred to as anchor residues [8,9]. Ca^2+^-CaM target sequences have been classified on the basis of the spacing of these hydrophobic anchors [10].

The CaM-binding site in myosin light chain kinases is a well-characterized example of a “1-14” motif, as there are 12 amino acids between its anchor residues at the first and fourteenth positions [11,12], whereas the kinase CaMKII typifies a “1-10” motif with 8 amino acids separating the anchor residues [13] (Figure 1). Both of these peptides are helical whereas the “1-16” CaM binding region of CaMK kinase contains a helix and hairpin that reduces the spatial distance between the anchors, which are more widely spaced in sequence [14]. These are referred to canonical CaM target motifs, however structures have been solved of CaM binding to many other non-canonical sequences. The flexible hinge of Ca^2+^-CaM allows it to stretch to bind helical ‘‘1-17′’ and “1-18” motifs RyR1 and PCMA pump and there are few contacts between lobes in these extended CaM structures [15,16,17] (Figure 1).

Remarkably, Ca^2+^-CaM can also adapt to bind a short “1-3” motif in myristoylated alanine-rich C kinase substrate (MARCKS) (Figure 1). This region, which is a non-myristoylated internal peptide, is unstructured with both anchors displayed on a single helical turn. One of these anchors is bound in the hydrophobic pocket of the C-lobe; however, the N-lobe is closed and its hydrophobic surface interacts with the other anchor and the C-lobe [18]. Interestingly, there are examples in which each lobe of Ca^2+^-CaM binds an anchor in separate polypeptides; CaM induces dimerization of glutamate decarboxylase (GAD) in this manner [19] (Figure 1). In the interaction between Ca^2+^-CaM and calcineurin (CaN), no anchor residues insert into the hydrophobic pockets of CaM, rather three hydrophobic residues interact with the edges of both hydrophobic pockets [20].

In the absence of Ca^2+^, apo-CaM interacts with several proteins that contain a so-called ‘IQ motif’ of the consensus sequence IQXXXRGXXXR, and many of these proteins also interact with Ca^2+^-bound CaM. Apo-CaM interacts with IQ motifs in cyclic nucleotide phosphodiesterase (PDE1A2), myosin V and the Na_V_1.2 and 1.5 channels primarily through a partially open C-lobe, with additional contacts via a closed N-lobe in some cases [21,22,23,24] (Figure 1).

CaM is a remarkably flexible and malleable protein, which can adapt its structure into distinct conformations to recognize and regulate a wide variety of target proteins.

### 1.2. Mechanisms by Which Ca^2+^-CaM Regulates Target Proteins

Binding of Ca^2+^-CaM to a target protein can lead to activation or inhibition of the target. Among diverse mechanisms of target activation [7], the best-known mechanism involves Ca^2+^-CaM binding releasing auto-inhibitory interactions. This is how Ca^2+^-CaM-dependent protein kinases (i.e., CaMKs) are regulated [25]. In the absence of Ca^2+^, the substrate binding site in the kinase domain is blocked by an auto-inhibitory region, which is adjacent to a CaM-binding site. A Ca^2+^ signal induces binding of Ca^2+^-CaM which leads to displacement of the auto-inhibitory peptide (Figure 1). Ca^2+^-CaM regulates the phosphatase CaN by a similar mechanism [8].

Binding of Ca^2+^-CaM to a target protein can also induce allosteric structural changes that can ‘remodel’ an active site [7]. The bacterial toxin edema factor is an adenylyl cyclase that is inactive until it is bound by CaM in a host cell. The Ca^2+^-bound C-lobe and Ca^2+^-free N-lobe bind distinct sites, which leads to stabilization of the adenylyl cyclase catalytic site [26] (Figure 1). CaM interacts with the Ras-association (RA) and pleckstrin homology (PH) domains of the adaptor protein growth factor receptor-bound protein 7 (Grb7) in a calcium-dependent manner and regulates its subcellular localization [27,28]. It was proposed that CaM binding may open the Grb7 structure, facilitating the RA and PH domains to interact with activated RAS and the membrane, respectively.

As described for GAD above, CaM can induce dimerization of target proteins through binding of each subunit to one of the lobes. Many ion channels are regulated by CaM in this manner as well. For example, Ca^2+^-CaM stimulates small conductance Ca^2+^-activated potassium (SK) channels through formation of a 2:2 complex in which the N- and C-terminal lobes each bind a different subunit [29]. Recent cryogenic electron microscopy (cryoEM) structures of other types of channels have revealed how they can be regulated by non-canonical interactions with CaM that either plug the pore or pull it open (reviewed in this issue [30]). Ca^2+^-CaM modulates expression of genes controlled by estrogen receptor α (ER-α) by inducing ER-α dimerization in a similar manner [31,32]. Ca^2+^-CaM also competes with ER-α for binding to a nuclear localization sequence in tuberin, thus there is a complex interplay between these proteins, which is not completely understood functionally [33].

In addition to these mostly helical CaM targets entirely comprised of polypeptide sequences, CaM has been reported to bind to peptides or proteins with polybasic ‘tails’ that are generally predicted to be unstructured and have myristoylated or prenylated terminal residues.

## 2. CaM Binding to Myristoylated Proteins

**Figure 2:**
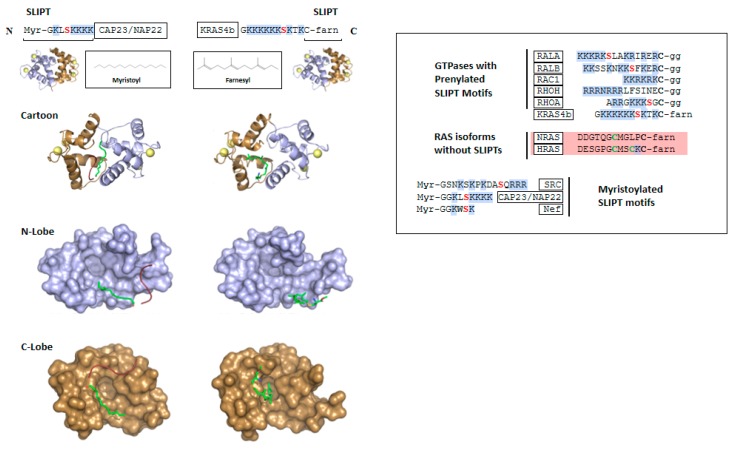
The Singly Lipidated Polybasic Terminus (SLIPT) non-canonical calmodulin-binding motif. Top: sequence of the myristoylated SLIPT motif in CAP23/NAP22 and the farnesylated SLIPT in KRAS4b. Middle: Ribbon models of Ca^2+^-calmodulin in complex with a myristoylated N-terminal peptide of CAP23/NAP22 (PDB accession code 1L7Z) and farnesyl cysteine methyl ester (FCME), representing the farnesylated C-terminal residue of KRAS4b (PDB 6OS4). The calmodulin C-terminal and N-terminal lobes are coloured brown and blue, respectively, with lipids in green and peptidyl moieties of the targets in red. Bottom: Space filling models of the N-terminal and C-terminal lobes of calmodulin showing how the SLIPT motifs interact. Right panel: Sequences of SLIPT motif sequences from KRAS4b, other small GTPase proteins and myristoylated proteins. Sequences of RAS isoforms lacking SLIPT motifs are also shown. Reported phosphorylation sites are red, palmitoylation sites are green and basic residues are highlighted in blue. Myr, Myristoyl; farn, farnesyl; gg, geranylgeranyl.

Ca^2+^-CaM has been reported to bind to several proteins that contain myristoylated polybasic N-terminal sequences. Around two decades ago, interactions between CaM and N-terminal myristoylated peptides were characterized biophysically, including the brain acid soluble protein 1 (referred to as CAP23/NAP22), and the viral proteins negative factor (Nef) from HIV1 and the oncogenic pp60^v-src^ tyrosine protein kinase from Rous sarcoma virus [34,35,36]. Nef and pp60^v-src^ localize to the plasma membrane in viral-infected cells, as does the cellular homolog c-Src, which is similarly myristoylated. Hayashi et al. used NMR to observe the binding of ^15^N Ca^2+^-CaM to an 8-mer myristoylated peptide derived from Nef and a longer 17-mer myristoylated peptide from pp60^v-src^, which contain 3 and 6 basic residues, respectively [35,36] (Figure 2). Both peptides induced substantial chemical shift perturbations in the heteronuclear single quantum coherence (HSQC) nuclear magnetic resonance (NMR) spectrum of ^15^N CaM, and these changes were remarkably similar, suggesting both peptides bind in a similar mode, although no NMR structures were determined.

Shortly thereafter, Matsubara et al. solved a crystal structure of Ca^2+^-CaM in complex with a myristoylated N-terminal polybasic peptide from CAP23/NAP22 [37]. The conformation of CaM bound to this myristoylated peptide differs from CaM in complex with any of the canonical peptides but does resemble CaM in complex with the MARCKS peptide. The N- and C-terminal lobes of CaM interact with each other forming a compact conformation with a hydrophobic core and acidic surface (Figure 2). The 14-carbon myristoyl moiety, which is saturated and unbranched, sits in a linear orientation in a hydrophobic channel formed by the hydrophobic surfaces of both lobes, but does not insert in the hydrophobic pockets that are occupied by the anchor residues of canonical Ca^2+^-CaM targets. The myristoyl moiety is attached to the N-terminal glycine residue of the CAP23/NAP22 polybasic sequence, a 9-mer containing 5 lysine residues. Electron density was detected only for part of the peptidyl component, but this was enough to infer that electrostatic interactions between the target lysine side chains and the acidic surface of CaM stabilize the interaction.

Ca^2+^-CaM binding to Src induces dissociation from the membrane [38]. If CaM interacts with the myristoylated Src N-terminus in a manner similar to CAP23/NAP22, the structure of the latter indicates that the sequestration of the lipid and potentially the polybasic residues is likely the underlying mechanism.

## 3. KRAS4b

Over the last two decades, interactions between CaM and KRAS4b have been reported by several groups, although there have been some discrepancies regarding the nature of the interaction. *KRAS* is of tremendous interest in oncology because *KRAS* mutations occur in 22% of all tumours, including 61% of pancreas, 33% of colon and 17% of lung cancers [39], 3 of the 5 most lethal cancers world-wide [40]. There are no drugs that target most KRAS mutant proteins, and these tumours respond poorly to chemotherapies. KRAS is a small GTPase protein that regulates signaling cascades like a switch. The protein adopts an activated state upon binding a molecule of GTP and is turned “off” by hydrolysis of GTP, which is impaired by oncogenic mutations. Activated KRAS can interact with and activate several effector proteins that drive cellular growth and proliferation. KRAS lacks the binding pockets that are typically targeted in classical drug discovery; however, inhibitors are available for many of the downstream kinases activated by KRAS signaling.

KRAS4b comprises a guanosine triphosphatase (GTPase) domain and a flexible C-terminal tail referred to as the hypervariable region (HVR) because it is not conserved amongst RAS isoforms. The C-terminus of KRAS4b contains a polybasic region and a CaaX box motif (C, cysteine; a, aliphatic; X, any residue) that is prenylated with a branched 15-carbon farnesyl group, and this post-translational modification is a requirement for KRAS4b signaling, which occurs on the plasma membrane [41,42]. Since the first report by Villalonga et al. in 2001, the literature has consistently shown that CaM interacts in a Ca^2+^-dependent manner with the RAS isoform KRAS4b, but not the KRAS4a, HRAS or NRAS isoforms [43,44,45,46,47]. Sidhu et al. demonstrated that CaM altered the partitioning of KRAS4b from membrane to cytosolic fractions [48], suggesting a mechanism by which CaM could inhibit KRAS4b signaling. The features that distinguish KRAS4b are: (i) its C-terminus is highly basic and (ii) it contains only a single lipidation site, whereas in addition to farnesylation, the other isoforms have one or more palmitoylation sites. The KRAS4b C-terminus has some propensity to form a helix [49], however, it lacks the hydrophobic anchor residues of the canonical CaM targets. Most, but not all studies have reported that CaM binding to KRAS4b requires farnesylation [43,44,46,50,51,52,53,54,55], and there are discrepancies in the literature about whether or not the GTPase domain or the nucleotide to which it is bound (GTP versus GDP) plays a role in the interaction.

### Structural Characterization of CaM Binding to KRAS4b

There have been a number of studies performed to elucidate the nature of CaM binding to KRAS4b. Not surprisingly, earlier studies focused on probing the direct interaction between CaM and the GTPase-domain of KRAS and proposed this interaction is GTP-dependent [43,45,50,51]. On the other hand, other researchers reported negative evidence for this interaction and concluded that KRAS G-domain does not bind CaM [44,46,51,53,54]. Recent in vitro biophysical and structural studies of the KRAS4b-CaM interaction [54,55] improved our understanding of this controversial interaction and revealed the detail of the CaM interaction with KRAS4b, which involves the farnesylated tail for binding to CaM.

NMR analyses of the interactions between KRAS4b, in both the GDP- and GTPγS-loaded states, and CaM in the presence and absence of Ca^2+^, clearly showed that binding is Ca^2+^- and farnesylation-dependent but does not require the KRAS4b GTPase-domain or the binding of a specific nucleotide [55]. CaM binding to farnesylated KRAS4b caused large chemical shift changes and severe line broadening, which is indicative of an interaction, but frustrated attempts to characterize the interaction. Remarkably, farnesyl cysteine methyl ester (FCME), representing the farnesylated C-terminal residue of KRAS4b, and farnesol alone, an alcohol derivative of the farnesyl moiety, induced a similar pattern of extensive Ca^2+^-dependent chemical shift perturbations in ^15^N CaM without causing line broadening [55]. Consistently, addition of a farnesylated polybasic 6-mer peptide from the KRAS4b C-terminus produced nearly identical changes in the CaM spectrum [54].

On the basis that NMR findings suggested CaM binding to FCME induces the same structural rearrangements as binding of farnesylated KRAS4b, CaM was crystallized in complex with this ligand producing a 1.8 Å crystal structure in which the two CaM lobes associate with each other to form a globular conformation with a hydrophobic core and the farnesyl moiety binds deeply in the hydrophobic pocket of the C-terminal lobe [55] (Figure 2). The cysteinyl moiety emerges between α-helices 1, 6, and 7, which would place the polybasic KRAS4b HVR proximal to an acidic region on the surface of CaM.

Structural data show that the KRAS4b-CaM interaction is principally mediated through sequestration of the KRAS4b farnesyl group, likely further stabilized by electrostatic contacts between the polybasic KRAS4b HVR and the acidic CaM surface [55]. Ca^2+^-CaM therefore sequesters the hydrophobic KRAS4b membrane localization moiety, and KRAS4b association with Ca^2+^-CaM was reported to be ~10-fold tighter than with the membrane [54,56]. Consistently, surface plasmon resonance (SPR) experiments with a lipid bilayer membrane captured on the biosensor chip showed that Ca^2+^-CaM extracted KRAS4b from this bilayer in a nucleotide-independent manner [46], and the transfer of prenylated KRAS4b between vesicles was accelerated by Ca^2+^-CaM [57]. The effects of CaM binding on KRAS4b association with a lipid bilayer was also investigated by NMR. KRAS4b NMR signals were broadened by association with a lipid bilayer (in the form of a nanodisc); however, the addition of Ca^2+^-CaM restored KRAS4b resonances that matched CaM-bound KRAS4b in the absence of lipids, again demonstrating that Ca^2+^-CaM extracted KRAS4b from the lipid bilayer [55]. Surface plasmon resonance (SPR) and isothermal titration calorimetry (ITC) experiments using isolated lobes of CaM demonstrated that both the N- and C-lobes are capable of binding KRAS4b, with the C-lobe exhibiting higher affinity [54]. Another approach using NMR analyses of CaM mutants in which either the N- or C-lobe cannot bind Ca^2+^, also demonstrated binding of farnesyl compounds to both the N- and C- lobes of CaM [55]. Consistently, analytical ultra-centrifugation (AUC) experiments showed that each molecule of CaM is capable of binding two farnesylated KRAS4b molecules in vitro [54], presumably with one molecule in each lobe. Similarly, small-angle X-ray scattering (SAXS) experiments showed that CaM can bind two molecules of myristoylated Src [58], however since the myristoyl group interacts with the surface of both lobes (in the CAP23/NAP22 structure [37]), it is not yet clear how the second molecule would be accommodated. In the cell, the stoichiometry of these complexes will probably depend on the ratio of free Ca^2+^-CaM to the lipidated targets, and their relative affinity to CaM versus the membrane or other interactors.

## 4. Comparison of CaM Bound to Myristoylated versus Farnesylated Moieties

FCME binding to ^15^N CaM induced chemical shift perturbations extremely similar to those induced by myristoylated polybasic peptides in Hayashi’s reports [35,36] and the FCME-bound CaM structure is very similar to that of CaM in complex with the myristoylated CAP23/NAP22 peptide [37,55]. The main distinction between these structures is that the saturated, unbranched myristoyl moiety extends through a hydrophobic channel formed by both lobes, whereas the branched farnesyl moiety inserts into the hydrophobic pocket of the C-terminal lobe, and the orientation of α-4 is slightly different. The C-terminal farnesylated cysteine sits in the same location as the N-terminal myristoylated glycine, thus the adjacent polybasic sequences should emerge from the hydrophobic core in the same place, although the direction of the peptides would be opposite. The conformation of CaM bound to the myristoyl and farnesyl moieties is distinct from the canonical peptide complexes but resembles CaM in complex with the largely unstructured internal ‘1-3′ motif from MARCKS [18].

Interestingly, a structure has been reported of Ca^2+^-CaM bound to a sphingolipid, with no peptidyl moiety attached [59]. CaM adopted a collapsed conformation with open hydrophobic pockets forming a hydrophobic channel that accommodated four molecules of sphingosylphophorylcholine, with its alkyl chains arranged in a parallel orientation. This interaction inhibited CaM by competing with its protein targets.

## 5. CaM Regulates KRAS4b Localization in Cells

In 2005, Fivaz and Meyer reported Ca^2+^-CaM dependent translocation of fluorescently tagged KRAS4b off the plasma membrane in hippocampal neurons with unknown mechanism [44]. The prenylated C-terminal tail of KRAS4b but not HRAS was reversibly internalized from the membrane to the cytoplasm and full length KRAS4b was similarly internalized regardless of the presence of constitutively activating or inactivating mutations (i.e., predominantly GTP- or GDP-bound, respectively). Most reports have been consistent that Ca^2+^-CaM extracts KRAS4b from lipid bilayers in vitro and in vivo, however they have differed on which elements are required [44,46,48,50,53,54,55]. Recently, a fluorescent biosensor for imaging the KRAS4b-CaM interaction to probe these questions in live cells [55].

**Figure 3:**
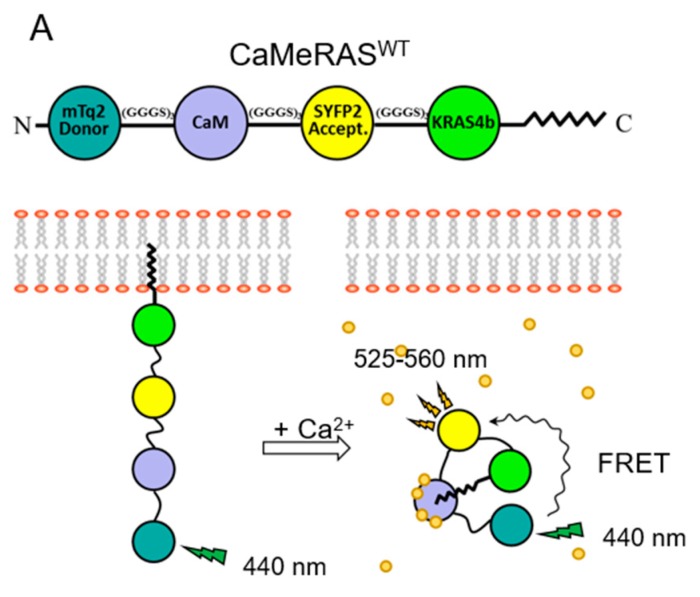

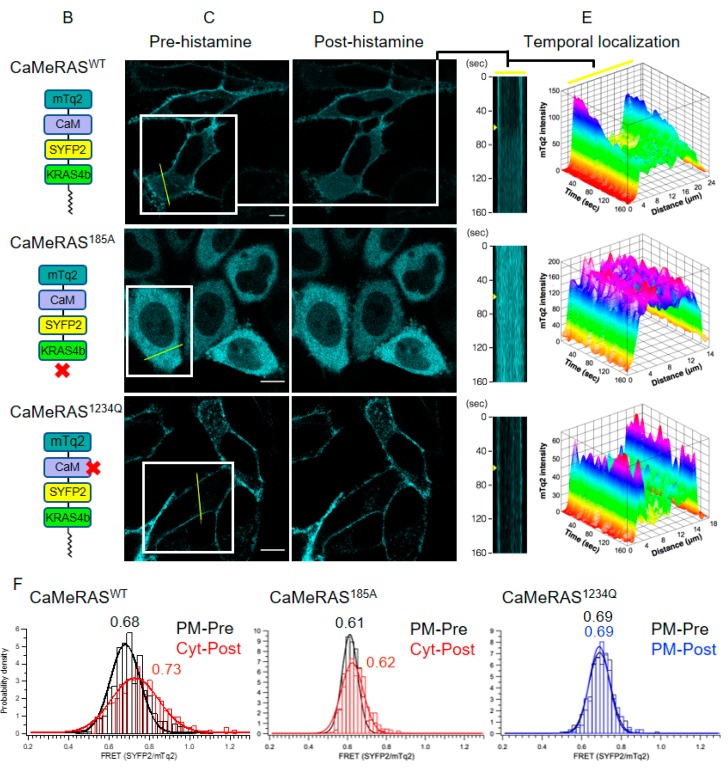
CaMeRAS demonstrates reversible Ca^2+^-/farnesyl-dependent internalization and FRET change. (**A**) Schematic of CaMeRAS and bimodal use for CaM-KRAS4b binding model validation. When transiently expressed in mammalian cells, the chimeric construct is natively processed at the C-terminal CaaX box (i.e., farnesylation of Cys185, cleavage of aaX residues, and carboxymethylation of the new C-terminus), allowing tethering of the construct to the plasma membrane. Upon cytosolic Ca^2+^ elevation, Ca^2+^-CaM extracts KRAS4b from the membrane, visible with fluorescence imaging, and the structural rearrangement of the chimera leads to detectable FRET. (**B**) Schematic of CaMeRAS variants transiently expressed in each experiment: CaMeRAS^WT^ (comprised of wild-type CaM and KRAS4b), CaMeRAS^185A^ (KRAS4b Cys185 mutated to Ala to prevent farnesylation), and CaMeRAS^1234Q^ (a key residue involved in coordination of Ca^2+^ in each of the four EF hands in CaM was mutated [i.e., E31Q, E67Q, E104Q and E140Q] to impair binding of Ca^2+^. An X indicates where each mutant is deficient. (**C**–**F**) After loading the cells with 10 µM Calbryte 630AM (AAT Bioquest), imaging was performed at room temperature using a Leica SP8 confocal microscope with a 63x/1.4 numerical aperture (NA) oil immersion objective lens and high-sensitivity HyD detectors. For simultaneous visualization of mTq2, SYFP2, and Calbryte 630, a 440-nm solid state laser and a white light laser tuned to 608 nm were used for excitation of mTq2 and Calbryte 630, respectively, and fluorescence emission was collected at 455 to 490 nm (mTq2), 525 to 560 nm (SYFP2), and 620 to 750 nm (Calbryte). Images were acquired at 3.4 to 4.0 s per frame. (**C**) Cellular localization (mTq2) of CaMeRAS constructs before histamine stimulation in HeLa cells. CaMeRAS^WT^ and CaMeRAS^1234Q^, farnesylated constructs, are localized on the plasma membrane, while CaMeRAS^185A^ is distributed in the cytoplasm. Scale bars measure 10 μm. (**D**) Cellular localization (mTq2) of constructs at 60 s after treatment with 100 μM histamine. The CaMeRAS^WT^ localization becomes cytoplasmic over time while CaMeRAS^185A^ and CaMeRAS^1234Q^ mutants retain their prior localization. (**E**) Representative mTq2 intensity distributions over time for cellular cross-sections (yellow lines in (**C**)) show changes in cellular localization following histamine stimulation at 60 s (yellow arrowheads) after starting imaging. CaMeRAS^WT^ experiences a shift from plasma membrane to cytosol while the membrane and cytosolic localization of CaMeRAS^185A^ and CaMeRAS^1234Q^ remain unperturbed. (**F**) FRET was measured by comparing the ratio of mTq2 to SYFP2 emissions within many small segments along the plasma membrane (PM) and in the cytoplasm (Cyt) before and after histamine stimulation. Histograms of FRET readouts (ratio of SYFP2/mTq2 emissions) pre- and post-stimulation. CaMeRAS^WT^ shows a significant change in FRET signal post histamine as it translocates from PM to Cyt (left). A minor FRET change was observed in Cyt for CaMeRAS^185A^ (middle), likely due the conformational change of CaM in response to Ca^2+^ binding. No FRET change was detected for CaMeRAS^1234Q^ (right). Data fitting the normal distributions was performed by using Gaussian functions. (Adapted from Grant et al. 2020; Reference [55]).

To observe the localization of KRAS4b and CaM and their interactions in cells, they were expressed as fusions with fluorescence resonance energy transfer (FRET) donor and acceptor fluorophores, however CaM overexpression was toxic. KRAS4b and CaM are both promiscuous proteins with many targets, thus they were fused together to promote intramolecular interactions over those with endogenous targets, which reduced toxicity. The chimeric biosensor ‘CaMeRAS’ comprises CaM flanked by the donor/acceptor fluorescent proteins mTurquoise2 (mTq2) and super yellow fluorescent protein 2 (SYFP2), and KRAS4b at the C-terminus to allow farnesylation (i.e., mTq2-CaM-SYFP2-KRAS4b) [55] (Figure 3A).

The premise of the CaMeRAS design was that the farnesylated C-terminus would localize it to the membrane under basal conditions, whereas a Ca^2+^ signal would induce Ca^2+^-CaM sequestration of the C-terminus, causing translocation to the cytosol and a structural rearrangement that may impact FRET efficiency (Figure 3A). Indeed, in vitro, Ca^2+^ increased the FRET efficiency of ‘wild-type’ CaMeRAS but not a mutant with impaired Ca^2+^ binding, whereas a farnesylation-deficient mutant exhibited a small change in FRET, probably due to the Ca^2+^-induced CaM structural change [55]. In vivo, CaMeRAS and the Ca^2+^-binding mutant localized to the plasma membrane, while only the wild type translocated to the cytosol in response to cytosolic Ca^2+^ elevation, and the farnesylation-deficient mutant was constitutively cytosolic [55] (Figure 3B–E). Further, changes in FRET efficiency (Figure 3F) and fluorescence-lifetime imaging (FLIM) were observed upon Ca^2+^-induced translocation of ‘wild-type’ CaMeRAS from the membrane to the cytoplasm [55]. This was observed in different cell types using different ligands to elicit the Ca^2+^ signal, which was simultaneously monitored with the fluorescent Ca^2+^-indicator Calbryte 630, confirming that CaMeRAS is a robust biosensor and supporting the model that Ca^2+^-CaM binding to the KRAS4b farnesyl group extracts it from the plasma membrane [55]. CaMeRAS is a multifunctional FRET biosensor, allowing simultaneous monitoring of both protein-protein interactions and translocation in cells.

## 6. Other RAS Isoforms and GTPases

Whereas KRAS4b has only a single lipidation site, the C-terminal regions of HRAS, NRAS, and KRAS4a undergo enzymatic cycles of palmitoylation/depalmitoylation at cysteine residues proximal to the site of farnesylation, which is irreversible (Figure 2). Palmitoylation strengthens anchoring to the membrane, and regulation of palmitoylation is a mechanism that modulates membrane attachment and controls signal output [60,61,62]. In contrast, KRAS4b membrane localization and thus signaling can be regulated through other mechanisms, including CaM in response to Ca^2+^ signaling, as well as phosphorylation of the polybasic region (described below). The other RAS isoforms have not been reported to interact with CaM, probably for multiple reasons. First, they lack the polybasic regions that likely mediate favourable electrostatics with CaM, and further, CaM may be incapable of competing with their enhanced membrane affinity.

Several other small GTPases have been reported to interact with CaM, including Rac1a, Rap1a, RalA, and RalB, all of which have SLIPTs but are geranylgeranylated rather than farnesylated at their polybasic C-termini [44,63,64,65,66,67,68,69] (Figure 2). It will be interesting to determine how the remarkable flexibility of CaM adapts to accommodate the 20-carbon geranylgeranyl moiety, which is longer than farnesyl (4 prenyl repeats compared to 3 in farnesyl).

## 7. A Plant CaM Isoform with a SLIPT

Nature has provided another intriguing example of how CaM binding a lipo-peptide moiety might modulate protein membrane attachment. Plants have many homologous CaM isoforms that exhibit significantly more variation in sequence than those in animals. The CaM isoform CaM53, found in petunia, has an unusual C-terminal extension that includes a polybasic region adjacent to a C-terminal farnesylation site [70]. This CaM protein cycles between the plasma membrane and nuclear localization with the diurnal cycle. The internalization of CaM53 was speculated to result from protein turnover and halted farnesyl synthesis, thereby preventing farnesylation of newly synthesized protein; however, plants respond to many external stimuli, including exposure to light and temperature, with tightly regulated Ca^2+^ influx [71,72]. It is therefore tempting to speculate that Ca^2+^ signaling may contribute to the cycle of CaM53 membrane-nuclear localization, potentially through Ca^2+^-induced intramolecular interactions between the farnesyl and the CaM domain, leading to extraction from the membrane in a manner analogous to CaMeRAS.

## 8. Regulation of CaM-Lipid Interactions by Phosphorylation of SLIPTs

The SLIPT motifs of several CaM-interacting proteins contain sites that can be phosphorylated, and they are often substrates of the ‘basophilic’ protein kinase C (PKC) family, due to the abundance of basic amino acids in the SLIPT [58]. Conventional PKC isoforms are activated by Ca^2+^ signaling, and phosphorylation of some of these substrates has been reported to disrupt CaM binding, although this interplay is not fully understood.

Interestingly, the N-terminal motif that specifies myristoylation (MGxxxSxx, after methionine cleavage) is an N-terminal glycine usually with a serine in the fifth position [58]. In CAP23/NAP22, this serine is phosphorylated, which abolishes interaction with Ca^2+^/CaM [34]. PKC phosphorylation of serine residues near the N-terminal myristoylation site in Src impairs binding of CaM [35,36], and the myristoylated N-terminus of HIV Nef1 can also be phosphorylated [58].

Analogous phosphorylation of the farnesylated C-terminus of KRAS4b has been well characterized. PKC phosphorylation of Ser181 proximal to the farnesylated C-terminus of KRAS4b reduces the positive charge on the polybasic region, which leads to redistribution from the anionic plasma membrane to more neutral endomembranes [73], where it interacts with inositol 1,4,5-trisphosphate receptors promoting autophagy and cell death [74]. CaM is antagonistic with PKC, as bound CaM protects the site from PKC phosphorylation, and conversely, phosphorylation disrupts CaM binding [44,50,57,75].

Canonical RAS signaling requires plasma membrane localization, thus the Ca^2+^-CaM-mediated translocation of KRAS4b to the cytoplasm inhibits these signaling pathways. At the same time Ca^2+^-CaM occupied by KRAS4b is unavailable to engage other target proteins, a corollary effect that has been reported to reduce the activation of CaMKII, which may promote tumorigenesis by limiting non-canonical Wnt/Ca^2+^ signaling [45].

In addition to KRAS4b, other putative GTPase CaM targets discussed above contain Ser/Thr residues adjacent to their prenylation sites. Rap1A, RhoA, RalA and RalB (Figure 2), have well-documented phosphorylation sites [76,77,78,79,80], and phosphorylation is unfavourable for interactions with the membrane and with CaM, analogous to KRAS4b.

## 9. Other Lipo-Peptide Sequestering Proteins

In addition to CaM, there are other examples of proteins that act to sequester lipid moieties of these myristoylated or prenylated proteins, through mechanisms that do not involve Ca^2+^ signaling, and do not require the presence of a polybasic motif. PDEδ, a noncatalytic subunit of the photoreceptor phosphodiesterase 6 (PDE6), binds the farnesyl moieties of small GTPases and other farnesylated proteins, but its role is not to extract them from the membrane. Rather PDEδ binding to the farnesylated C-terminus of KRAS4b in the cytoplasm has been proposed to be responsible for returning it to the plasma membrane through an active mechanism regulated by the Arf-like (ARL) GTPases [49,81,82,83]. PDEδ and CaM therefore would compete for binding to the farnesyl group of KRAS4b, and their effects on its localization seem to oppose each other. The affinity of CaM for KRAS4b is ~5-fold higher than that of PDEδ [49,54], although its interaction is limited to the duration of a Ca^2+^ signaling event. It appears that PDEδ and CaM functions are spatiotemporally coordinated, in that CaM extracts KRAS4b from the plasma membrane upon a Ca^2+^ signaling event, whereas PDEδ plays a role in restoring plasma membrane localization. Small molecules that inhibit PDEδ lead to delocalization of KRAS4b from the plasma membrane and impair its oncogenic signaling [84,85].

Small GTPases in the Rab and Rho subfamilies, which are geranylgeranylated, interact with guanine-nucleotide-dissociation-inhibitors, which, like PDEδ, sequester the prenyl group and solubilize these GTPases away from membranes [86]. Rab-escort-proteins solubilize newly prenylated Rab proteins and deliver them to the membrane [87].

In a system that is analogous to PDEδ transport of farnesylated proteins, myristoylated proteins can be solubilized by uncoordinated-119 (UNC119) [88], and their release is similarly triggered by ARL GTPase proteins [89]. Some myristoylated proteins possess regulated mechanisms to sequester their own lipid moieties in an intramolecular manner. For example, release of the myristoyl group and membrane localization is triggered by GTP binding in the ARF GTPases [90] and Ca^2+^ binding in recoverin [91].

## 10. Complex Interplay between CaM and Lipidated Targets, and Crosstalk between Ca^2+^ and Oncogenic Signaling

In addition to Ca^2+^-CaM binding the myristoylated N-terminus of Src, three other potential binding sites have been proposed, including two atypical IQ motifs, and while Ca^2+^-CaM stimulates the kinase activity of Src it also leads to its dissociation from the membrane, and apo-CaM has been proposed to be a more potent activator of Src [92]. Src can phosphorylate two Tyr residues of CaM, which alters its interactions with specific targets in different ways [93]. Src also phosphorylates two Tyr residues in the switch regions of KRAS4b, which disrupts its interactions with both effector and regulatory proteins (i.e., GTPase activating proteins, GAPs and guanine nucleotide exchange factors, GEFs) [94,95]. Additionally, Src kinase activity can induce Ca^2+^ signaling through its substrate proteins and KRAS4b is a Ca^2+^-dependent CaM-binding target. Many questions remain to be answered about the direct interplay between these proteins, let alone multiple indirect mechanisms of crosstalk.

These interactions between CaM, Src and KRAS4b are some of many examples of interplay between the Ca^2+^ and MAPK signaling pathways that are relevant to oncogenesis. In 1998, Bosch et al. observed that inactivation of CaM in serum-starved NIH3T3 cells causes activation of ERK1/2, MEK, C-Raf, and RAS, irrespective of receptor tyrosine kinase phosphorylation [96], and since then, a variety of mechanisms linking these pathways have emerged. For example, the Ca^2+^-dependent kinase PYK2 promotes recruitment of Grb2 and SOS to the plasma membrane where it activates RAS. Other RAS GEFs are also regulated by Ca^2+^: RAS protein-specific guanine nucleotide-releasing factors (RAS-GRFs) contain “IQ” CaM binding motifs and are regulated by Ca^2+^-CaM, while some of the RAS guanyl-releasing proteins (RAS-GRPs) have EF hands and are regulated directly by Ca^2+^ [97,98]. In addition to Ca^2+^-modulated GEFs, some RAS GAPs are regulated by Ca^2+^ as well: p120 RAS GAP and Ca^2+^-promoted RAS inactivator (CAPRI) both translocate to the plasma membrane in response to Ca^2+^ signalling. Thus, the GTPase cycle and localization of KRAS4b, both of which are required for canonical signaling, are influenced by Ca^2+^ signals. Reciprocally, feedback mechanisms exist by which RAS-MAPK activity modulates Ca^2+^ signaling proteins. Ca^2+^ signalling is dysregulated in many cancer cells, often through over- or under-expression of calcium channels (e.g., stromal interaction molecule 1, STIM1; Calcium release-activated calcium channel protein 1; Transient receptor potential channels; IP_3_ (inositol 1,4,5-trisphosphate) receptor; and L-type voltage-gated calcium channels, Ca_v_1) [99]. In some cases, this has been linked to transcriptional control by KRAS4b signalling. For example, deletion of an oncogenic KRAS allele or MEK inhibition results in enhanced STIM1 expression and store-operated Ca^2+^ entry [100]. The complex interplay between Ca^2+^ and RAS-MAPK signaling pathways requires further study.

## 11. Conclusions

Together the myristoylated and prenylated sequences with a ‘singly lipidated polybasic terminus’ (SLIPT) define a non-canonical Ca^2+^-dependent CaM target motif. These SLIPT motifs are crucial for membrane localization, which is often antagonized by the binding of Ca^2+^-CaM, through direct sequestration of the lipid moieties. The polybasic portion of these motifs frequently contain a serine that can be phosphorylated by PKC or other kinases, which often reduces the affinity of the motif for both the membrane and CaM. In addition to relief of autoinhibition, allosteric remodeling, mechanical modulation, and induced dimerization, disruption of membrane localization is an additional mechanism by which CaM regulates its target proteins.
